# Use and evaluation of assistive technologies for upper limb function in tetraplegia

**DOI:** 10.1080/10790268.2021.1878342

**Published:** 2021-02-19

**Authors:** Rosti Readioff, Zaha Kamran Siddiqui, Caroline Stewart, Louisa Fulbrook, Rory J. O’Connor, Edward K. Chadwick

**Affiliations:** 1 School of Pharmacy and Bioengineering, Keele University, Stoke-on-Trent, UK; 2 Academic Department of Rehabilitation Medicine, Faculty of Medicine and Health, University of Leeds, Leeds, UK; 3 The Orthotic Research and Locomotor Assessment Unit (ORLAU), the Robert Jones and Agnes Hunt Orthopaedic Hospital, NHS Foundation Trust, Oswestry, UK; 4 School of Engineering, University of Aberdeen, Aberdeen, UK

**Keywords:** Assistive technology, Tetraplegia, Spinal cord injury, Upper limb

## Abstract

**Context:**

More than half of all spinal cord injuries (SCI) occur at the cervical level leading to loss of upper limb function, restricted activity and reduced independence. Several technologies have been developed to assist with upper limb functions in the SCI population.

**Objective:**

There is no clear clinical consensus on the effectiveness of the current assistive technologies for the cervical SCI population, hence this study reviews the literature in the years between 1999 and 2019.

**Methods:**

A systematic review was performed on the state-of-the-art assistive technology that supports and improves the function of impaired upper limbs in cervical SCI populations. Combinations of terms, covering assistive technology, SCI, and upper limb, were used in the search, which resulted in a total of 1770 articles. Data extractions were performed on the selected studies which involved summarizing details on the assistive technologies, characteristics of study participants, outcome measures, and improved upper limb functions when using the device.

**Results:**

A total of 24 articles were found and grouped into five categories, including neuroprostheses (invasive and non-invasive), orthotic devices, hybrid systems, robots, and arm supports. Only a few selected studies comprehensively reported characteristics of the participants. There was a wide range of outcome measures and all studies reported improvements in upper limb function with the devices.

**Conclusions:**

This study highlighted that assistive technologies can improve functions of the upper limbs in SCI patients. It was challenging to draw generalizable conclusions because of factors, such as heterogeneity of recruited participants, a wide range of outcome measures, and the different technologies employed.

## Introduction

Each year in the UK, 1000 people sustain a traumatic spinal cord injury, and in total 40,000 people live with a spinal cord injury (SCI).^1–3^ This number is higher in the United States, where approximately 294,000 (range 250,000–368,000) individuals live with SCI and each year around 17,810 new SCI cases are reported.^4–6^ More than half of all cases of SCI occur at the cervical level leading to loss of hand and upper limb function.^6,7^ This complex impairment results in restricted activity and independence, hence significantly compromising wellbeing and quality of life.^8,9^ This life-changing injury remains a particular challenge to modern society as there is no cure. However, technological systems have been developed to restore some upper limb function for individuals with tetraplegia due to SCI including systems with neuroprostheses, orthotics, robots, and hybrid devices.

Individuals affected by high-level SCI see restoration of upper limb functions as a high priority.^10^ Increased motor function in the hand and arms for this population can be achieved by surgical interventions or by assistive technologies.^11,12^ Unlike therapeutic technologies, which seek to improve physical impairments, assistive technologies are designed to assist with the performance of specific tasks for the user and intended for use when neurological recovery has reached a plateau. There has been ongoing research and development on assistive technologies for tetraplegia in the last 20 years. There is no clear clinical consensus on the effectiveness of the current assistive technologies for the cervical SCI population; therefore, we decided to review the literature for the years between 1999 and 2019.

The aim of this study was to systematically review the state-of-the-art assistive technology that supports and improves function of impaired upper limbs in people with cervical SCI. In addition, clinical outcomes, resulting from the implementation of such technologies, have been reviewed. To fulfill the aim of the study, we set out two main objectives and they were to:

1. Describe the assistive technology, with a focus on devices that interface with the upper limbs; and
2. Describe the outcome measures used when testing the efficacy of the technologies.

## Methods

### Search strategy

An electronic search of databases, including (CINAHL, AMED, EMBASE, PUBMED, MEDLINE, EMCARE) from 1999 to 2019, was performed. Initially, three categories essential to assess assistive technologies for clinical purposes were established: clinical condition, type of technology, and affected body part. Combinations of search terms within the three categories were used, sometimes with truncation, to capture all possible variations (Table 1). Two examples of search strategies are shown in the supplementary materials (Example S1 and S2). In addition to the electronic search of the databases, the reference lists of relevant publications were checked.

**Table 1:** Included and excluded terms used for electronically searching databases.

	Clinical condition	Type of technology	Affected body part
Search terms for inclusion	Spinal cord injury, SCI, spinal cord lesion, tetraplegia, quadriplegia, tetraplegic, quadriplegic, paralysis	Assistive technology, assistive device, orthotic device, splint, robotics, arm support, mobile arm support, anti-gravity support, neuroprostheses, functional electrical stimulation, FES, neuromuscular electrical stimulation, NMES, hybrid device, neuromuscular electrical stimulation, arm-weight bearing, implanted electrical stimulator, surface electrical stimulator, percutaneous electrical stimulator.	Upper limb, upper extremity, hand, arm, forearm, forelimb
Search terms for exclusion	Stroke, multiple sclerosis, MS, polio, poliomyelitis, paraplegic, paraplegia	Prosthesis, prosthetics, exoskeleton, passive assistive device, artificial limbs	Lower limb, lower extremity, leg

### Study selection

Initially, duplicate, low-level of evidence (for example articles with excluded terms), and irrelevant articles were discarded. Subsequently, the remaining articles were assessed based on their title and abstract, and 10% of these articles were blindly re-assessed by another reviewer. With the 10% of article re-assessment we found little difference of opinion, hence giving us confidence in the selected articles. Agreement was reached by discussion and reasoning in case of discrepancies. Following abstract and title screening, full texts of the articles were reviewed for final screening.

### Data extraction

The main categories for data extraction were type of assistive technology and its description, study participants, outlines of outcome measure, and functional ability with and without assistive technology. This information was used to summarize the efficacy of the current assistive technology for the upper limb in populations with tetraplegia.

## Results

### Study selection

**Figure 1:**
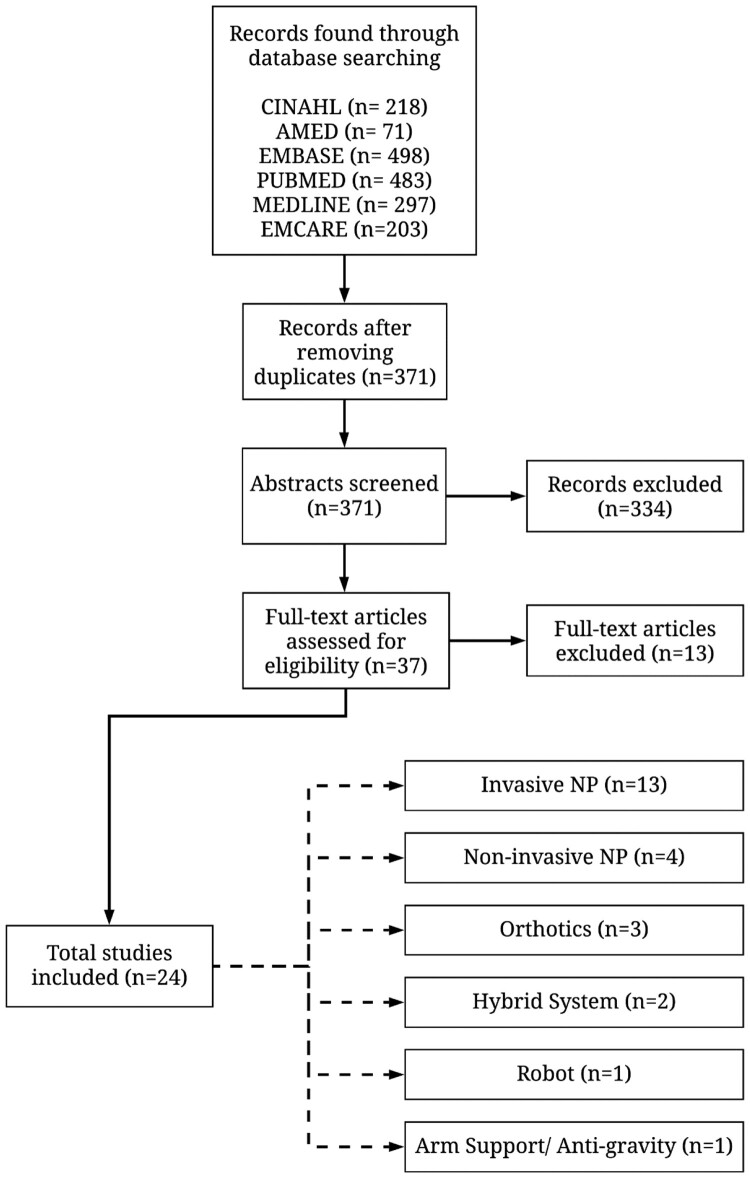
Study selection flow diagram for the searched databases. Abbreviations: NP, neuroprostheses.

The literature search in CINAHL, AMED, EMBASE, PUBMED, MEDLINE, and EMCARE yielded 218, 71, 498, 483, 297, and 203 studies, respectively. Following the initial study selection process, 371 studies were found. Subsequently, the abstracts of these studies were screened by searching for the predefined inclusion and exclusion terms (Table 1). Abstract screening yielded 37 studies. The 37 studies were further assessed for inclusion in the current study by reading the full text of the articles while looking for contents relevant to assistive technologies for the upper limb in cervical SCI population, and a clear report on outcome measures. The full-text assessment resulted in selecting a total of 24 studies for the analysis in this paper (Fig. 1). Of the 24 selected studies, 13 were identified as case studies or series,^13–25^ two as clinical trials,^26,27^ one as a clinical study,^28^ and eight as cohort studies.^29–36^

### Data extraction

#### Identified assistive technologies

In this study, assistive technologies for restoring upper limb function in populations with spinal cord injury were categorized as follows:

- Neuroprosthesis (invasive^20–26,29–34^ and non-invasive ^18,19,27,28^) is a system where muscles are stimulated by small electrical currents to generate motor functions;
- Orthosis is a non-invasive supportive device which assists with optimum use of remaining motor control;^16,35,36^
- Hybrid system is a combination of multiple technologies such as neuroprosthesis and orthosis,^13,14^ or powered orthosis;
- Robot is a non-invasive device generating functional movements without the need for users to have any residual motor control,^17^ and;
- Antigravity arm support is an add-on device to other assistive technologies.^15^

From the literature search, 20 of the selected studies focused on neuroprostheses, with sixteen on invasive and four on non-invasive neuroprostheses. Selected studies, focusing on assistive technologies other than neuroprostheses, were limited, with three on orthotics, two on hybrid systems, one on robots, and one on antigravity arm supports. Descriptions of the identified assistive technologies are reported in Table 2.

**Table 2:** Descriptive summary of assistive devices in the selected studies. Abbreviations: FES, Functional Electrical Stimulation; BCI, Brain–Computer Interface; IST-12, 12-channel implantable stimulator-telemeter; MES, Myoelectric Signals; EMG, Electromyographic.

Authors	Type of assistive device	Device description
Thorsen *et al.* ^28^	Non-invasive neuroprosthesis	A one-channel battery-powered portable neuroprosthesis implementing myoelectric controlled functional electrical stimulation (MeCFES). Standard surface self-adhesive stimulation electrodes and EMG recording electrodes were used and connected to the MeCFES unit by flexible cables.
Alon and McBride^18^	Non-invasive neuroprosthesis	The Handmaster as described in Snoek *et al*. (2000). The control unit enables the user to choose between three exercise and three functional modes which are key grip and release, palmar grasp and release, and static open hand posture.
Snoek *et al.*^19^	Non-invasive neuroprosthesis	The Handmaster is a neuroprosthesis combining a spiral wrist and hand orthosis with integrated surface electrodes to activate muscles of the paralyzed forearm and hand. Three exercise and two functional modes can be selected on the control unit. The functional modes provide, key grip and release, and palmar grasp and release, while exercise modes provide repetitive stimulation of the muscles.
Popovic *et al.*^27^	Non-invasive neuroprosthesis	The Bionic glove uses three channels of electrical stimulation to stimulate finger flexors, extensors, and thumb flexors. The control signal comes from a wrist position transducer mounted in the garment.
Bockbrader *et al.*^20^	Invasive neuroprosthesis	A BCI system comprising of a 96-channel Utah microelectrode array implanted in the left dominant cortex, which interfaces with transcutaneous forearm FES.
Kilgore *et al.*^29^	Invasive neuroprosthesis	IST-12 described in Kilgore *et al*. (2008).
Friedenberg *et al.*^21^	Invasive neuroprosthesis	A BCI system interfacing a Utah microelectrode array implanted in the left primary motor cortex with the FES technology. The FES system of a multi-channel stimulator flexible cuff, consisting of up to 140 electrodes, is wrapped around the subject’s arm.
Memberg *et al.*^22^	Invasive neuroprosthesis	IST-12 described in Kilgore *et al*. (2008).
Gan *et al.*^23^	Invasive neuroprosthesis	Stimulus Router System (SRS) is a neuroprosthesis device in which only passive leads are implanted on branches of the upper limb nerves and each lead picks up a portion of the current transmitted through the skin by an external stimulator. User triggers stimulation with small tooth-clicks that are detected by the wireless earpiece containing a 3-axis accelerometer. Wristlet stimulators are used to generate trains of pulses that are delivered through 4 electrode pads. Three of these pads are located over the 3 pick-up terminals and the fourth centered 12 cm proximal to the wrist crease on the posterior aspect of the forearm.
Kilgore *et al.*^30^	Invasive neuroprosthesis	IST-12 is a second-generation implantable neuroprosthesis to control hand grasp, forearm pronation, and elbow extension. This device has the capacity to stimulate 12 paralyzed muscles and record MES from two muscles under voluntary control. Device is controlled through implanted MES recording electrodes and MES processing circuitry. The system is driven from an external power and processing unit, which are connected to a coil that the participant places on the skin over the implanted device.
Mangold *et al.*^24^	Invasive neuroprosthesis	Neuroprosthesis comprising of transcutaneous self-adhesive electrodes which delivered FES via a stationary stimulation system and two portable systems (ETHZ- ParaCare FES system and Complex Motion). The control sensor varied between subjects from a digital push button switch, EMG signals, and sliding potentiometer (analogue control).
Memberg *et al.*^31^	Invasive neuroprosthesis	An elbow extension neuroprosthesis previously reported by Bryden *et al*. (2000).
Taylor *et al.*^32^	Invasive neuroprosthesis	The Freehand system described in Carroll *et al*. (2000).
Peckham *et al.*^33^	Invasive neuroprosthesis	The Freehand system described in Carroll *et al*. (2000).
Yu *et al.*^25^	Invasive neuroprosthesis	A percutaneous intramuscular stimulator restoring elbow and shoulder functions without stimulating muscles of the hands. The device comprises of implanted electrodes in the shoulder and elbow muscles. The device is controlled via a switch on the headrest of the user’s wheelchair and a position sensor on the contralateral shoulder. Weak stimulated shoulder movements were compensated for by adding a forearm orthosis.
Carroll *et al.*^26^	Invasive neuroprosthesis	The Freehand system, an implanted 8-channel neuroprosthesis device, providing unilateral hand grasp and release. The device comprises of implanted and external components. The receiver–stimulator, epimysial electrodes, and inter-lead connectors are implanted internally, whereas the external components are a controller, a transmitter, and a sensor at the shoulder. The neuroprosthesis is controlled using contralateral shoulder movements (either protraction–retraction or elevation–depression).
Bryden *et al.*^34^	Invasive neuroprosthesis	An elbow extension neuroprosthesis consisting of fully implanted tricep electrodes. This intervention is implemented as an addition to the Freehand System. Hand grasp was controlled using a shoulder or a wrist controller. Stimulation of the triceps for elbow extension was controlled using a switch or an accelerometer on the user’s upper arm or applying a constant level of triceps stimulation when the hand grasp stimulation is active.
Portnova *et al.*^35^	Orthosis	A personalized three-dimensional printed wrist-driven orthosis comprising of 11 parts: hand, forearm, palmar and dorsal pieces, long and short bars, input link, thumb and finger pieces, and two finger rings.
Kang *et al.*^36^	Orthosis	A personalized wrist-driven flexor hinge orthosis (WDFHO) consisting of a polyethylene forearm and a palmar cuff to grasp objects. The device stabilizes the index and middle fingers along with the interphalangeal and metacarpophalangeal joints of the thumb. The device pushes together the thumb, index, and middle fingers when the wrist is extended and releases the fingers when the wrist is flexed.
King *et al*. ^16^	Orthosis	A lateral key grip orthosis comprising of a flexible cable running along the anterior surface of the forearm to the palmar region of the hand, further attaching to a ring around the thumb proximal phalanx. Tension on the cable pulls the thumb into palmar adduction so that a grip forms against the lateral region of the proximal or middle phalanx of the index finger.
Rohm *et al.*^13^	Hybrid System	A modular hybrid device consisting of a combination of FES with orthoses and BCI controller. The orthosis has anti-gravity module to support elbow flexion and extension during stimulation of triceps. The device comprised of a wrist-stabilizing module to keep the wrist in neutral position enabling finger flexion. To facilitate the FES, a personalized neoprene sleeve, with defined electrode positions, was manufactured. The device was controlled using a motor imagery BCI and an analogue shoulder position sensor.
Varoto *et al.*^14^	Hybrid System	A hybrid device comprising of a glove that combines orthosis with forearm support along with neuromuscular electrical stimulation. While the elbow dynamic orthosis with forearm support allows elbow flexion and extension, static orthosis supports the wrist and neuromuscular electrical stimulation generates grasping function. The glove with force sensors also allows grasping force feedback via two user interface modes: visual by light emitting diodes or audio emitted by buzzer.
Cappello *et al.*^17^	Robots	A fabric-based soft robotic glove combined with modular, independent finger actuators attached by straps, hook, and loop fasteners. Each actuator is comprised of three fabric layers and two air-tight bladders between each fabric pocket, one for flexion and the other for extension. The glove is controlled by a portable and self-contained control box with three buttons performing a finger flexion and extension, 3-point pinch, and palmar grasp.
Asai and Kuroiwa^15^	Antigravity arm support (i.e. portable spring balancer (PSB), and mobile arm support (MAS))	Two devices reported: A portable spring balancer consisting of three metal parts: an aluminum tube containing a spring to assist the arm in resisting gravity; a proximal arm connected to the aluminum tube allowing vertical movement; and a steel bar connecting the distal arm assembly to a distal cuff, supporting the arm at the elbow and wrist. A mobile arm support consisting of a distal arm assembly, a proximal arm assembly, a trough, and a bracket. The device was mounted on the subject’s wheelchair.

#### Study participants

Characteristics of the participants recruited into each study are summarized in Table 3. Not all of the selected studies comprehensively reported characteristics of their participants, for example two studies did not report participants’ sex,^14,28^ two studies did not report participants’ age,^16,31^ and five studies did not report time between injury and participant recruitment.^14,20,28,31,34^ In twenty-two studies, the neurological level of the injury ranged from C4 to C8, and two were above C3. The time since injury varied widely (range from 3 months to 62 years) with no particular pattern or correlation to the assistive devices in the selected studies.

**Table 3:** Descriptive summary of study participants in the selected studies. Abbreviations: M, Male; F, Female; NR, Not Reported.

Authors	Type of assistive device	Participant number, Sex	Participant age in years* Range (Median)	Time since injury in years** Range (Median)	Lesion at
Thorsen *et al.*^28^	Non-invasive neuroprosthesis	27, NR	18–80 (NR)	NR	C5–C7
Alon and McBride^18^	Non-invasive neuroprosthesis	7, M	25–46 (37)	3.1–17.3 (11.2)	C5–C6
Snoek *et al.*^19^	Non-invasive neuroprosthesis	10, M (8) and F (2)	20–65 (30.5)	0.5–6 (1)	C4–C6
Popovic *et al.*^27^	Non-invasive neuroprosthesis	12, M	18–38 (22)	0.25–2 (2)	C5–C7
Bockbrader *et al.*^20^	Invasive neuroprosthesis	1, M	27	NR	C5
Kilgore *et al.*^29^	Invasive neuroprosthesis	12, M (10) and F (2)	26–56 (37.8)	1–21 (3.8)	C5–C6
Friedenberg *et al.*^21^	Invasive neuroprosthesis	1, M	27	6	C5–C6
Memberg *et al.*^22^	Invasive neuroprosthesis	2, M and F	27 and 48	1.1 and 11	C1–C3
Gan *et al.*^23^	Invasive neuroprosthesis	1, M	52	14	C6/C7
Kilgore *et al.*^30^	Invasive neuroprosthesis	3, NR	24–43 (34)	1–4 (2)	C5–C7
Mangold *et al.*^24^	Invasive neuroprosthesis	11, M (9) and F (2)	15–70 (32)	1–62 (1)	C4–C7
Memberg *et al.*^31^	Invasive neuroprosthesis	10, M (9) and F (1)	NR	NR	C5–C6
Taylor *et al.*^32^	Invasive neuroprosthesis	9, M (8) and F (1)	NR (Mean = 38.4)	NR (Mean = 10.1)	C5–C6
Peckham *et al.*^33^	Invasive neuroprosthesis	51, M (42) and F (9)	16–57 (32)	1.1–32.2 (4.6)	C5–C6
Yu *et al.*^25^	Invasive neuroprosthesis	1, M	24	3	C3
Carroll *et al.*^26^	Invasive neuroprosthesis	6, M (4) and F (2)	21.9–36 (30.1)	1.2–11.3 (2.7)	C5–C6
Bryden *et al.*^34^	Invasive neuroprosthesis	4, M	23–48 (33)	NR	C5–C6
Portnova *et al.*^35^	Orthosis	3, M (2) and F (1)	40–65 (54)	16–28 (18.5)	C4–C6
Kang *et al.*^36^	Orthosis	24, M (22) and F (2)	NR (37.1 ± 12.8) ^+^	NR (5.6 ± 7.3) ^+^	C6–C7
King *et al.*^16^	Orthosis	7, M	NR	0.5–4 (0.5)	C5–C7
Rohm *et al.*^13^	Hybrid System	1, M	41	1	C4
Varoto *et al.*^14^	Hybrid System	5, NR	29–42 (36)	NR	C5–C8
Cappello *et al.*^17^	Robots	9, M (8) and F (1)	20–68 (53)	0.4–44 (33)	C4–C7
Asai and Kuroiwa^15^	Antigravity arm support	4, M	15–50 (19)	1.2–0.3 (0.8)	C4–C5

* Participant age range indicates the age of participant at the time of recruitment for the study.

** Time since injury indicates the time between injury and recruitment for the study.

^+^ Mean ± SD.

#### Outcome measures

The outcome measures, adopted in the selected studies, covered a variety of the domains that comprise the framework of International Classification of Functioning, Disability and Health (ICF).^37^ In total, there were 30 different outcome measures assessing body functions and structures, activity, and participation domains (Supplementary Materials Table S1). In the body functions and structure domain, outcome measures described joint movement, force generation, active and passive range of motion (ROM) through a number of standardized tests, such as Jebsen-Taylor-Hand-Function (JTHF) and Toronto Rehabilitation Institute Hand Function Test (TRI-HFT). In the activity domain, outcome measures were evaluated using a range of tests, including, Grasp-and-Release-Test (GRT), Activity of Daily Living (ADL), Action Research Arm Test (ARAT), Functional Independence Measure (FIM), and Spinal Cord Independence Measure (SCIM). In the participation domain, outcome measures assessed individuals when using the device in the community through tools and surveys, including the Craig Handicap Assessment and Reporting Tool (CHART). Only one study clearly reported on this domain, investigating social integration and occupation subscale,^30^ and three studies carried out satisfaction surveys and participant questionnaires for using the device at home.^24,33,34^

#### Study functional outcomes

All studies reported improvement in functional ability of the upper limb while using the assistive devices (Supplementary Materials Table S1). Studies on neuroprostheses, both invasive and non-invasive devices, showed increased hand function, grip and pinch strength, average range of movement in the upper limb, and improvement in ADLs.

In one study, the application of non-invasive neuroprostheses showed an immediate increase in hand function in 63% of their compliant subjects of whom 15% scored a clinically relevant change of 5.7 ARAT points.^28^ Studies reported that grip strength was increased from 0.57N to 16.5N,^18^ average range of movement in the forearm and wrist was increased by 9%,^27^ and participants successfully performed at least three new ADL tasks.^18,19,27^ Participants, who continuously used the non-invasive neuroprosthesis devices, showed a 75% higher performance of the ADL tasks.^27^ Similarly, the effect of training with the device increased ARAT score by 2 points which is clinically important.^28^ In addition, using non-invasive neuroprosthesis is thought to cause therapeutic effects and improve hand function.^28^

Participants, with invasive neuroprostheses, had undergone invasive methods to implant the device. The implanted components of the device consist of epimysial and intramuscular electrodes, electrode leads, and electromyography recording electrodes.^22,29,30^ Some studies combined corrective surgeries such as tendon transfer with invasive neuroprostheses to further improve upper limb function.^24,30,32,33^ Participants, using invasive neuroprostheses, were able to manipulate objects with varied size, surface, and weights.^18,20,24,26,29,30,32,33^ For example, GRT scores showed that 92% of participants improved the ability to manipulate objects,^29^ participants at least doubled the number of objects manipulated or tasks performed,^30,32,33^ and lateral and palmar grasp improved.^33^ A study, combining assistive technology with corrective surgeries, such as arthrodesis, tendon transfers of muscles, and tendon synchronization, reported pinch force values at three stages (before intervention, after corrective surgery, and after surgery with assistive device).^30^ Pinch force was increased from 4 N before to 12 N after corrective surgery and then to 19 N with device use, in other words pinch force was increased by 58% after surgery with device use.^30^ Increase in pinch force, when using the device, was also reported in lateral, palmar, and finger grasps.^26,32^ The range of lateral pinch force with the device was 11.6 N to17 N,^22,29,32,33^ palmar pinch force was 6.5 N to 10.4 N,^29,32,33^ and finger grasp was 14.7 N.^32^ The improvement of grasp and release function and strength of grips contributed to the increased success of the ADL tasks. ADL tasks, reported in the selected studies, varied widely, some studies allowed participants to choose the ADL tasks^24,30,32^ and others predefined an extensive list of the tasks.^13,15,18,19,22,26,27,33,34^ The results from ADL tests showed that participants experienced reduced disability and increased independence when using the invasive-neuroprosthesis.

Pinch force in participants increased with the use of an orthosis, such that in one study pinch force with an orthosis was 14.3 times greater than without the device.^36^ Another study found that an orthosis increased maximum voluntary contraction (MVC), resulting in an increase of lateral grip force (the force ranging between 4.7 N and 22.3 N).^16^ Only one study looked at the effect of an orthosis on performing ADL tasks, and reported that a greater number of tasks were achieved with the device compared to without.^16^

Studies on hybrid systems reported successful performance of GRT tasks,^13^ and increased ability to manipulate objects using palmar grasp.^14^

The only study evaluating the antigravity arm support device on its own showed that the device facilitates ADL tasks such as eating.^15^ A combination of mobile arm supports with other assistive devices, such as neuroprostheses, to support the weight of an arm has been reported but not evaluated on their own.^22^

## Discussion

In this review, we defined a set of inclusion and exclusion criteria to systematically select original research articles, focusing on the state-of-the-art assistive technologies, which support and improve function of impaired upper limbs in cervical SCI populations. The objectives of the paper are fulfilled by describing the assistive technologies and the outcome measures used to assess them.

During study selection, it was noted that a larger number of recent studies focused on developing control systems to regulate assistive technologies for the upper limbs.^38–47^ Similarly, several studies reported the use of rehabilitation technologies, such as training and therapeutic tools to restore function in the upper limb.^48–59^ These studies were excluded in this review paper so that a comprehensive focus could be made on the efficacy of assistive technologies that offer ongoing support to the upper limb for restoring function in people with cervical-level SCI. As a result of the study selection, the assistive technologies developed, trialed, or used for restoring upper limb function were grouped into five categories, namely, neuroprostheses, orthoses, hybrid systems, robots, and antigravity arm supports. Some of the technologies described can be assigned to multiple categories. The Handmaster, for example, is a combination of a hand orthosis with surface electrodes and it is categorized under non-invasive neuroprosthesis.^18,19^ One reason for placing the Handmaster in the non-invasive neuroprosthesis category is because the studies investigated the neuroprosthesis more than the orthotic part of the device. The same reasoning was used for other devices that spanned categories, such as antigravity arm support devices and neuroprosthesis.^15,22^ However, devices with multiple technologies, such as those reported in,^13,14^ are classified as hybrid systems. A survey study, involving participants with SCI, reported that many of the participants were not aware of the current assistive technologies, hence they were not aware of available options that could improve their independence and quality of life.^60^ It is possible that the lack of clear and accessible categories of assistive technologies for restoring the upper limb functions could have been a factor.

The incidences of SCIs vary across countries, regions, and cities. A study reviewing global prevalence of SCI highlighted that the highest SCI prevalence was in the US (Alaska), while the lowest prevalence was in France (Rhone-Alpes region).^61^ Globally, there was a greater percentage of males with SCI than females.^61^ The demographics of recruited participants in the selected studies showed a high male-to-female ratio, such as 11:1, 9:1, and 8:1,^31,32,36^ and a wide age range between 19 and 54 years old. Others recruited either one participant or a relatively lower male-to-female ratio, such as a ratio below 5:1. In the UK, for the years between 1985 and 1988, male-to-female ratio for people, sustaining a spinal cord injury, was 3.8:1 with an average age of 35.5 and 46 years old for males and females, respectively.^62^ No more recent consensus about the epidemiology of SCI in the UK was found, however, the demographics of SCI patients in the developed countries is believed to have changed in terms of age more than sex over the last 20 to 40 years.^6,61,63,64^ In the US, for the years between 1970 and 2015, the average age at injury increased from 29 to 43 years.^6^ Similarly, in Scotland, for the years between 1994 and 2013, there was a notable increase of new SCI in the over 50-year old population and those with high level (C1–C4) tetraplegia.^63^ With the exception of Japan, where SCI patients in their 70s are the largest age group,^65^ a larger percentage of SCI patients are under the age of 30 in most countries.^61^ The assistive technologies were population dependent and inclusion criteria for participant recruitment was focused more on injury level than age of participants or time since injury. Two of the selected papers recruited participants with C3 or higher levels of injury and the assistive technologies implemented were invasive neuroprostheses.^22,25^ Participants in these two studies had limited to no voluntary contraction in the upper or lower limbs, hence it is possible that the decision on the type of device for this population was based on practicality for device operations. Similarly, participants in studies investigated orthotic devices for SCI had the ability to extend their wrist against gravity.^16,35,36^ It would be beneficial for future studies to outline reasons behind opting to use an assistive device, so that a library of different types of assistive devices and their suitability for different SCI populations can be established.

Prior to adopting an assistive device, SCI patients go through a rehabilitation process which commences in the acute care setting and lasts for 6 to 12 weeks, during this time the focus is on patient’s neurological stability status, indirect complications, such as pressure ulcers, maintaining range of motion and preventing muscle atrophy.^66^ Early rehabilitation is believed to prevent the development of joint contractures, especially contractures of elbow flexion and supination.^67^ Therefore, identifying a suitable assistive technology to meet the needs of SCI patients at the early rehabilitation stage might improve the efficacy of the selected assistive device, hence enhance the patients’ quality of life. The literature showed limited focus on the relationship between the efficacy of assistive technologies and time since injury. It was reported that assistive technologies built for functional purposes have therapeutic effects, however, small to no significant correlation was reported between time since injury and functional outcomes as a result of the device use.^28^ The selected literature assessed functional capabilities of the assistive devices through clinical outcome measures.

The outcome measures for assessing the assistive technologies were in the activity domain of the ICF. A limited number of the selected studies covered all three domains of the ICF; however, all covered the activity domain. All studies, except for three, followed the outcome measures identified by the Spinal Cord Injury Research Evidence (SCIRE) to assess the effect of device use on activities.^14,21,34^ One of the three studies, investigating a hybrid system, assessed shoulder and scapular movements when using the device through the measurement of rotational speed, real-time angular variation, and real-time force. Although these measurements indicated an increased palmar grasp, the study did not clearly report the translation of the measurements and their relevance to ICF activity domain.^14^ Similarly, a study on invasive neuroprosthesis reported an improved volitional control across a continuous wrist angle but did not test the effect of this increased ability on participant’s activities.^21^ The third study focused on elbow extension using invasive neuroprosthesis, developed new evaluations to assess the device because at the time the existing tests did not evaluate specific functions.^34^ They reported that specific information (i.e. interval data) to augment the muscle grade is needed because few gradations exist for a muscle that achieves full ROM and takes resistance. To obtain interval instead of ordinal data, they developed a technique of measuring the weight against gravity when participants were extending their elbows. Grip or pinch strength measurement was the most frequently used outcome measure after ADL tasks. Interestingly, this finding aligns with the choices professional practitioners make when selecting an outcome measure from SCIRE toolkit during their practice.^68^ Professionals tend to choose the SCIM and FIM for assessing self-care and daily living, the GRT for assessing the upper limb functions, and the Quebec user evaluation of satisfaction and predisposition assessment for assessing the effect of assistive technology. The outcome measures reported in this review included the FIM, SCIM, GRT and Graded Redefined Assessment of Strength, Sensibility, and Prehension (GRASSP) and these were reported to be reliable and valid.^69–71^ Whereas the Quadriplegia Index of Function (QIF) is suggested for use only in non-ambulatory tetraplegia and its validity has not been investigated sufficiently.^69^ It is important to note that, unlike the FIM, the SCIM, GRT, GRASSP, and QIF were specifically designed for the SCI population. The FIM was designed to assess a broad range of disabling medical conditions, hence it might not specifically reflect on measures for SCI population. In addition, the selected papers in this review were limited by the lack of assessment on the efficacy of the assistive technologies during mobility. It is essential for future studies to assess assistive devices by looking at function, activity, and independence in the context of mobility from the ICF.

There are challenges and limitations that come with utilizing assistive technologies for people with SCI. Loss of proprioception, for example, can make it challenging for people with SCI to adopt the aforementioned assistive technologies.^72^ Other disadvantages of assistive technologies, such as invasive neuroprostheses, include risks associated with surgical operations and potentially additional surgeries to reposition migrated electrodes or replace failed hardware components. However, compared to non-invasive neuroprostheses’ stronger forces and better muscle selectivity can be achieved with invasive neuroprostheses because the stimulation electrode can be implanted closer to the motor nerve and in deeper muscles.^73–75^ In addition, the orthosis, robots, hybrid systems, and antigravity arm supports are advantageous because of their non-invasive nature; however, they are disadvantaged by the difficulties with donning, doffing, and achieving selective muscle stimulation.^76^ In addition to the generic disadvantages of these assistive devices, the technologies reported in the selected literature were limited, including the fabric-based soft robotic glove which could not generate adequate pinch grasp between the thumb and index finger due to a deficiency in their actuator design.^17^ Furthermore, a study on grasp coordination with an invasive neuroprosthesis did not have an electrode to stimulate thenar muscle; therefore, they could not accurately measure the maximal palmar, lateral, and tip-to-tip grip force.^20^ It is worth mentioning that achieving the upper limb movements with assistive devices alone can be challenging; therefore, a number of the studies reported a combination of surgical and technological interventions for improving upper limb functions.^24,30,32,33^ For example, corrective surgeries, such as tendon transfer, were performed to augment the system.^32^ A study reported that smaller objects, such as pegs and wooden blocks, could be manipulated better with an active tenodesis grasp rather than with a transcutaneous functional electrical stimulation.^24^ This is because the position of the object within the hand can be corrected more effectively and there is no time required for the interaction with the device. However, the electrical stimulation is advantageous and sometimes necessary to manipulate heavier or slippery objects. Assistive technologies, combined with corrective surgeries, could provide higher degrees of upper limb functionalities in tetraplegia.^33,73^ Further research is needed to investigate the efficacy of assistive technologies when they are integrated with corrective surgeries, such as tendon^77^ or nerve transfer.^78^

In conclusion, here we categorized the assistive technologies into five main cohorts, hence making the evidence base of current technology more accessible and identifiable for clinicians, users, researchers, and readers. There is evidence that the assistive technologies reported in this study can help people living with cervical SCI. Compared to the other technologies, a larger number of studies focused on the development of neuroprostheses two decades ago which was followed by much less interests in recent years. As a result, the application of neuroprostheses has been more extensively studied recently, hence future research is equipped to focus on developing user–control systems. There is an imbalance on how the efficacy of assistive technologies is assessed in relation to the three domains of the ICF. We recommend future studies on assistive technologies to follow the outcome measures identified by SCIRE and, when possible, equally address the three domains of the ICF in order to better quantify the effectiveness of assistive technologies. For example, future studies could focus on developing and following a methodology that would facilitate comparisons between different assistive devices.

## Supplementary Material

Supplemental Material
