# Supplementary material for: Use and evaluation of assistive technologies for upper limb function in tetraplegia
Source: J Spinal Cord Med. 2021 Feb 19;45(6):809–20. doi: 10.1080/10790268.2021.1878342 (PMC9662059; doi:10.1080/10790268.2021.1878342)
Supplement: Supplemental Material [file YSCM_A_1878342_SM5271.docx]

**Use and evaluation of assistive technologies for upper limb function in tetraplegia**

Supplementary Materials

**A. Search Strategy Examples for two databases**

**Example S1: Medline**

Medline: (((exp "SELF-HELP DEVICES"/ OR exp "ORTHOTIC DEVICES"/ OR exp SPLINTS/ OR exp ROBOTICS/ OR exp "EXOSKELETON DEVICE"/ OR exp "ELECTRIC STIMULATION"/ OR exp "TRANSCUTANEOUS ELECTRIC NERVE STIMULATION"/ OR ("self-help device*" OR "self help device*" OR "assistive technology" OR "assistive device*" OR "orthotic device*" OR orthotic* OR splint* OR robotic* OR "arm support*" OR "anti-gravity support*" OR "antigravity support*").ti,ab OR (neuroprosthe* OR "functional electrical stimulat*" OR FES OR "neuromuscular electrical stimulat*" OR NMES OR "hybrid device*" OR "arm-weight bearing" OR "implanted electrical stimulat*" OR "surface electrical stimulat*" OR exoskeleton OR "percutaneous electrical stimulat*").ti,ab) AND (exp QUADRIPLEGIA/ OR (quadripleg* OR tetrapleg*).ti,ab)) AND (exp FORELIMB/ OR exp ARM/ OR exp "UPPER EXTREMITY"/ OR ("upper limb" OR "upper extremity" OR hand OR arm OR forearm OR forelimb OR brachium* OR antebrachium* OR "membrum superius").ti,ab)) [DT FROM 1999] [Languages English]"

**Example S2: CINAHL**

CINAHL: (((exp QUADRIPLEGIA/ OR (exp "SPINAL CORD INJURIES"/ AND exp "CERVICAL VERTEBRAE"/) OR (exp "CERVICAL CORD"/ AND (inur* OR lesion*).ti,ab) OR (quadripleg* OR tetrapleg* OR "cervical spinal cord injur*" OR "cervical spinal cord lesion*" OR (spinal cord lesion* AND cervical) OR (spinal cord injur* AND cervical)).ti,ab) AND (exp "UPPER EXTREMITY"/ OR exp ARM/ OR exp HAND/ OR exp FOREARM/ OR ("upper limb*" OR "upper extremit*" OR hand* OR arm* OR forearm* OR forelimb* OR brachium* OR antebrachium* OR "membrum superius").ti,ab)) AND (exp "ASSISTIVE TECHNOLOGY DEVICES"/ OR exp "ASSISTIVE TECHNOLOGY"/ OR exp ORTHOSES/ OR exp "EXOSKELETON DEVICES"/ OR exp SPLINTS/ OR exp ROBOTICS/ OR exp "ELECTRICAL STIMULATION, FUNCTIONAL"/ OR exp "ELECTRICAL STIMULATION, NEUROMUSCULAR"/ OR exp "TRANSCUTANEOUS ELECTRIC NERVE STIMULATION"/ OR ("self-help device*" OR "self help device*" OR "assistive technology" OR "assistive device*" OR "orthotic device*" OR orthotic* OR splint* OR robotic* OR "arm support*" OR "anti-gravity support*" OR "antigravity support*").ti,ab OR (neuroprosthe* OR "functional electrical stimulat*" OR FES OR "neuromuscular electrical stimulat*" OR NMES OR "hybrid device*" OR "arm-weight bearing" OR "implanted electrical stimulat*" OR "surface electrical stimulat*" OR exoskeleton OR "percutaneous electrical stimulat*").ti,ab)) [DT FROM 1999] [Languages eng]"

**B. Tables**

**Table S1:** Overview of assistive devices in the selected studies, their corresponding outcome measures and participant’s functional ability with and without using the device. Abbreviations: NP, Neuroprosthesis; GRT, Grasp and Release Test; JTHF, Jebsen Taylor Hand Function; MVC, Maximum Voluntary Contraction; TRI-HFT, Toronto Rehabilitation Institute Hand Function Test; MRC, Medical Research Council; CHART, Craig Handicap Assessment and Reporting Tool; QIF, Quadriplegia Index of Function; FIM, Functional Independence Measure; UEFT, Upper Extremity Function Test; GRASSP, Graded Redefined Assessment of Strength, Sensibility, and Prehension; ARAT, Action Research Arm Test; CUE-T, Capabilities of Upper Extremity Test; QIF-SF, Quadriplegia Index of Function-Short Form; SCIM-SR, Spinal Cord Independence Measure-Self-Report.

***Blue****: Non-invasive neuroprosthesis.* ***Green****: Invasive neuroprosthesis.* ***Yellow****: Orthosis.* ***White****: Hybrid system.* ***Grey****: Robot.* ***Purple****: Antigravity arm support.*

|  | **Outcome Measure** | **Functional Ability without Assistive Device** | **Functional Ability with Assistive Device** |
| --- | --- | --- | --- |
| **Thorsen *et al.* (2013)** | - ARAT - Proportions of subjects having positive change scores and the proportions exceeding the clinically relevant improvement. | - Wrist extension above grade 1 on the MRC scale. | - The immediate effect increasing hand function in 63% of the compliant participants and 15% of which exceeded the clinically relevant change of at least 5.7 ARAT points. Positive correlation of baseline ARAT with immediate effect was observed. - The intervention period using this device caused therapeutic effect and improved the hand function in 56% of the participants. - After training with the device, 67% of participants demonstrated an increase in the ARAT score by 2 points which was clinically important. |
| **Alon & McBride (2003)** | - Hand impairment tests:  1. Grip strength. 2. Distance of finger motion from the distal palmar crease to the fingertip of the most extended finger 3. Fugl-Meyer hand subset of spherical grasp (scored 0=cannot perform, 1=ball kept in the hand against slight tug, and 2=ball is held against firm tug).  - Three ADLs (pick up a telephone, eat food with a fork, perform 1 individually selected task, and 2 grasp, hold and release tasks). | - Adequate passive ROM of the fingers and thumb and a spasticity grade of less than 2. However, no grasp and release hand function and no finger motion. - The grip strength was 0.57 ± 0.98 N. - One out of seven was able to score 1 (hold the ball) without the device. - Results of the ADL showed 21% successful attempts. | - Significant improvements occurred in grip strength (16.5 ± 4.4 N), finger linear motion (8.4 ± 3.2cm), and Fugl-Meyer scores. - All participants successfully performed both the grasp, hold and release tasks, and were able to perform the task on the first attempt 92% of the time. - All participants successfully performed the three ADL tasks. |
| **Snoek *et al.* (2000)** | - Five ADLs (pouring water from a can, opening a jar, opening a bottle, taking a video tape out and putting it into a video player, one task selected by participant) | - Two participants lacked shoulder movement. - None of the participants could perform ADLs such as handling objects, pouring liquids, brushing, cutting, putting socks on, dry shaving. | - All participants were able to perform several tasks with the device. - Two participants were able to use the key as well as the palmar grasp mode for functional tasks, while the other two participants were only able to use the palmar grasp. |
| **Popovic *et al.* (1999)** | - Tonus of forearm and hand muscles using passive movement of fingers and flexible goniometers. - QIF - FIM - UEFT (11 tasks: combing hair, using a fork, picking up a VCR tape, picking up a full juice can, picking up a full pop/soda can, writing with a pen, answering the phone, brushing teeth, pouring from a 1 litre juice box, drinking from a mug, and handling finger food). - Weekly log forms. | - All participants had active wrist extension ranging from 10° to 45° and wrist extension strength 1+ to 5+ measured on a scale of 0 to 5. - Most of the participants lacked active extension and flexion of thumb and fingers, and flexion of the wrist. - Three participants had active wrist flexion to 40°. - One participant had active thumb extension and flexion to full range. - One participant had active finger flexion and extension to full range. | - Average range of movement increased from 2% to 11% when using the device. - The mean QIF increased by 49.5% (from 19.0 ± 6.5 to 28.4 ± 5.2). After 6 months of using the device only 36% of the maximum possible value was achieved. - The mean FIM value for all participants increased from 63.8 ± 10.4 to 79.0 ± 8.9 after 6 months. - 75% of ADL tasks were better performed in participants who continued using the device. |
| **Bockbrader *et al.* (2019)** | - Box and Block Test - GRASSP - ARAT - GRT - CUE-T - QIF-SF - SCIM-SR | - The participant had full strength during shoulder and elbow flexion, with limited wrist extension. - GRASSP strength was 24% of normal strength. - Prehension ability score based on ability to grip was 42% of normal. - Prehension performance was 30% of normal. - The total ARAT, grasp and grip were 32%, 44% and 33% of normal scores. - CUE-T total score was 45% of normal. - QIF-SF baseline score of the participant was 4. - SCIM-SR baseline score of the participant was 15. | - GRASSP strength improved to 80% of normal, achieving normal strength for five forearm muscle groups. - Prehension ability 92% of normal score. - Prehension performance improved to 50% of normal score due to better ability to pour a bottle, unscrew lids and perform 9-Hole peg. - Manual dexterity improved total ARAT (53% of normal), grasp (83% of normal) and grip (75% of normal) scores. - GRT success rate improved for all objects except for block. - Box and block test showed no improvements in transfer rates. - CUE-T total score improved to 82% of normal. However, no change was noted in reaching, lifting, pushing or pulling scores. - QIF-SF showed an increase in participant’s expected level of independence for ADLs (QIF-SF scored 13). - SCIM-SR showed an increase in participant’s level of independence (scored 24) for self-care and toileting and limited independence for bed mobility. |
| **Kilgore *et al.* (2018)** | - ROM - Grip strength - GRT | - No subject had any active moment in their fingers or thumb - Only one participant could manipulate as many as four out of six objects. | - Stimulation produced active extension and flexion for all five digits in all participants, with total ROM between 105° to 41°. - There was a significant difference in grasp strength for both lateral (~17 N post-implant with device) and palmar (~6.5 N post-implant with device) grasps. - All participants manipulated the same number of objects. In addition, three participants manipulated one extra object and one of the participants manipulated two extra objects. |
| **Friedenberg *et al.* (2017)** | - Neural activity measured by mean wavelet power - Cue angles measured on a protractor | - Complete paralysis of hand and wrist. | - Participant successfully hit the target angles in the set of three training blocks at a rate of 90.3 ± 3.5% (mean ± standard error), where success was defined as maintaining position within a ±15° window around the target cue angle for at least two continuous seconds. - Participant was able to volitionally control own graded muscle contraction across a continuous range of angles. The participant achieved an accuracy of 89.6 ± 4.4% including successes on 14 out of 18 cues for angles that had not been attempted previously. - Participant sustained flexion of paralysed wrist and hand, and reliable pointing at the target angles with an average success rate of 88.9 ± 3.7%. |
| **Memberg *et al.* (2014)** | - ROM (wrist, forearm, elbow, and shoulder) - Grip strength - Joint moments - Six ADLs (eating with a fork and finger food, hand shaking, nose scratching, nose wiping with a tissue, face washing, and teeth brushing). | - Participants had complete motor paralysis in at least one upper extremity. - Neither subjects were able to support the weight of their arm with stimulation of their shoulder muscles alone. | - Shoulder abduction moments evoked by suprascapular and axillary nerve stimulation showed large increase. Stimulation of the musculocutaneous nerve activated the biceps and brachialis, producing elbow flexion. Radial nerve stimulation activated two muscles at the elbow: triceps causing extension, and brachioradialis causing flexion. The elbow flexion and extension moments were sufficient for moving the arm to perform ADL. - Stimulated lateral pinch strength ranged from 11.6 to 25.5 N in the two subjects. - ROM at the shoulder: flexion ranged from 47° to 74°, adduction ranged from 3° to 8°, abduction ranged from 25° to 27°, internal rotation was 64°. - ROM at the elbow: extension 0°, flexion ranged from 88° to 109°. - ROM at the forearm: pronation ranged from 0° to 15°, supination ranged from 18° to 57°. - ROM at the wrist: extension was -10°, flexion was 18°. - ADLs: - Feeding with a fork - success depended on spasticity level on the day; - Eating finger foods - successful with some help; scratching nose - successful; - Wiping nose with tissue - success with some help; - Washing face with washcloth - somewhat successful with help; - Brushing teeth - somewhat successful with help; and - Shaking hands - successful. |
| **Gan *et al.* (2012)** | - Maximal grip strength - Thumb-finger aperture (hand-opening measurement) | - Unable to generate a measurable grip force on the dynamometer. - Voluntary flexion at the wrist achieved an aperture of hand opening of 4 cm between the tips of the forefinger and thumb. - A maximal grip force of 29.6 N was achieved with a 30/s train of pulses (300ms, 19mA) applied to the flexors. | - Mean maximal grip force ranged from 50 to 100 N in 100 days post-implant. - 14 cm thumb-finger separation was achieved during maximal stimulation after 4 months which was 4cm greater than the separation achieved with surface FES prior to surgery. |
| **Kilgore *et al.* (2008)** | - Body functions and structures: - Grasp - pinch force - Activities: - GRT - ADLs (feeding, grooming, writing and other activities specified by participant) - Participation: - CHART | - Pinch force was ranging from 2 to 11 N for all three participants. - Grasp release test performance showed that two out of three participants were able to complete peg, block and can tasks. - Participants were unable to perform activities they were set out to do with the NP device. | - All participants improved their pinch force strength such that one participant demonstrated an increase in pinch force from 4 N before surgery, to 12 N after tendon transfer surgery (without device use) and then to 19 N (with device use). - All participants were able to double the number of objects manipulated in the grasp and release test. Two out of three participants were able to complete all six tasks for this test. - All participants improved in at least five activities with all demonstrating improvement in eating with a fork, drinking from a glass, and writing. Two of the three participants improved performance in 5 out of 11 tasks and one participant improved performance in 9 out of 11 tasks. - All participants demonstrated increased scores for the physical independence subscale. No changes in social integration were noticed in two of the participants and one participant increased their social integration subscale. One participant showed improvement in mobility subscale whereas the other two showed a slight decrease in this subscale. Two of the participants reported a decrease in the occupation subscale and one reported no change. |
| **Mangold *et al.* (2005)** | - Sollerman test (11 tasks, including manipulating objects of different sizes, geometries, and complexities). - Self-designed ADL functional test focusing on grasp-and-release tasks. - Status of muscle strength - Follow up query assessing the applicability of device in hospital and at home. | - No active palmar or lateral grasp functions. - Sufficient proximal arm function. | - Two participants showed improvements in muscle strength and facilitation of active movement with the device. - Eight participants demonstrated improved grasp function and performance in ADLs. - Most improvement in grasp function was observed in those participants who were not able to grasp bimanually or had no tenodesis grasp. - All participants (11 in total) used the device during their training programme. Number of participants used the device for ADL in rehabilitation centre and at home reduced to four and two. |
| **Memberg *et al.* (2003)** | - Strength measurement: moments generated by elbow flexion at 30°, 60°, 90°, and 120° while shoulder was positioned at 90° abduction and 0° horizontal adduction (keeping the upper arm horizontal, and the elbow in line with both acromia). - Workspace Assessment: assessing the effect of triceps on the controllable workspace by having participants reach, grasp, and move a book-like object from high location or orientation to a low location or orientation. Success rates and acquisition times were recorded. | - Only one participant was able to extend their arms against gravity without stimulation, using self-triggering spasm that resulted in triceps activation. | - Average stimulated elbow extension moments for the 11 arms with the elbow at 90° flexion ranged from 0.8 to 13.3 Nm. - 8 of the 11 arms were able to extend against gravity with triceps stimulation. - The elbow moment generated by triceps stimulation at 90° and 120° elbow flexion was significantly greater than the elbow moment produced by the posterior deltoid tendon transfer. - Elbow extension moment by participants with posterior deltoid tendon transfer ranged from 0 to 11.2 Nm. However, no difference in elbow moment between the two elbow extension methods at 30° elbow flexion. - The quantitative workspace assessment was more successful with stimulation than without. Success rate varied from 15-61% and it was improved for all participant at both far and near locations, similarly for when the book oriented vertically. - Average acquisition times with triceps stimulation were less than without the stimulation for 4 out of 5 arms, such that improvement in average acquisition time ranged from 3.2 to 6.4s. |
| **Taylor *et al.* (2002)** | - GRT (wooden pegs, a 250 g weight, a plunger, wooden cubes, a plastic cylinder, a small juice can, a videotape). - Grip strength - Eight ADL tasks chosen by participant. | - Participants with C5 injury were unable to complete any GRT tasks. - Participants with C6 injury were able to complete some of the tasks involving light objects or small force. - Four of the participants had sufficient tenodesis grip with a mean lateral, palmar and five finger grasps of 0.93 N, 0.96 N, and 1.04 N, respectively. | - GRT score was improved when device was used, and participants performed on average 5.1 types of task (maximum 6). - The mean lateral, palmar and five finger grasps had increased to 15.2 N, 10.4 N and 14.7 N respectively at 1-year post training. - Most of the selected tasks were achieved in the ADL assessment indicating a significant improvement in independence. |
| **Peckham *et al.* (2001)** | - Pinch strength - Active ROM - GRT - ADL Abilities, and ADL assessment tests - Satisfaction survey | - Lateral and palmar pinch forces were 0.3 N and 0 N, respectively. - Participants were able to manipulate smaller and lighter objects. - 68% of the participants were able to demonstrate lateral grasp using a peg, whereas none of the participants could successfully manipulate a weight or a fork. - 57%, 16%, and 7% of the participants were able to demonstrate palmer grasp using a block, a can and a tape, respectively. | - Lateral and palmar pinch forces were increased to 12 N and 6.6 N, respectively - 98% of participant moved at least 1 more object with the neuroprosthesis and 37% improved by moving at least 3 more objects. - 100%, 90% and 86% of the participants demonstrated lateral grasp by manipulating a peg, a weight, a fork respectively. - 98%, 78%, 72% of the participants demonstrated palmar grasp by manipulating a block, a can and a tape, respectively. - Disability was reduced in all assessed participants as measured by either ADL Abilities or ADL Assessment Tests. - All assessed participants improved in independence in at least 1 task and 64% in at least 3 tasks. - Satisfaction survey showed that 97% of participants will recommend the device to others, and 91% state that the device improved their quality of life. |
| **Yu *et al.* (2001)** | - Stimulated active ROM (SAROM) - Self-feeding activities | - Fair voluntary shoulder elevation and retraction bilaterally. No other voluntary muscle contraction in the upper limbs. | - SAROM against gravity included 60° of shoulder abduction, 45° of shoulder flexion, 10° of shoulder external rotation with the shoulder passively abducted to 90°, and 110° of elbow flexion. - Stimulated elbow extension lacked 20° of full extension with gravity eliminated. - Subject was able to pick up mashed potatoes from a plate and bring them to his mouth. |
| **Carroll *et al.* (2000)** | - Pinch forces - GRT (peg, weight, fork, can, block and tape) - Eight ADLs (including using a telephone, drinking from a cup, brushing teeth and writing with a pen) | - No voluntary finger or thumb movements - Minimal or no wrist extension - Only light items (peg and block) could be manipulated due to passive tenodesis. | - Pinch forces, lateral and palmar show a substantial increase in force with the device. - All subjects were able to grasp, move and release more objects within the test period with the neuroprosthesis than without it. - Tenodesis function appeared to be improved in four subjects after implantation of the device. - During ADL tasks, participants required less assistance 73% of the time. - Improvements in hand function were seen in 97% of activities. |
| **Bryden *et al.* (2000)** | - ROM - Five ADLs (activating a slide dimmer light switch, activating an overhead pull-chain light switch, hanging a garment, using a wall-mounted paper towel dispenser, and acquiring and placing an object on a shelf). - A participant survey for home use of the device. | - All participants had passive elbow extension that was within normal limit. - No participant could attain full elbow extension against gravity. - Active elbow extension was either absent or less than full range without stimulated triceps for all participants even for those with the posterior deltoid to triceps transfer. | - All participants attained full elbow extension (i.e. to zero degrees). - All were able to abduct their shoulder by at least 29° while maintaining full elbow extension with triceps stimulation on versus off. - All participants reached their max shoulder abduction (i.e. to their passive limits) while maintaining full elbow extension. - All participants were able to extend the elbow against gravity repetitively without added weight, with the number of repetitions ranged from 12-43. All participants could resist at least 0.5 kg and one could resist almost 3.5 kg against gravity. - All participants showed improved functional performance in 92% of tasks. - Use of stimulated elbow extension reduced the amount of assistance that was required by 56%. - All participants required less assistance with stimulated triceps to perform at least one task. - Three of four participants (4 extremities) preferred to perform 100% of the tasks with stimulated elbow extension. - All found at least some of the tasks easier to perform with stimulated elbow extension. - Participants showed regular use of the device at their home and community. |
| **Portnova *et al.* (2018)** | - Box-and-Blocks test - JTHF test - Three-point pinch force | - All participants had little to no mobility in their fingers but were able to extend their wrists against gravity. | - Two out of three participants showed improvement during the Box-and-Block test and they managed to transfer more blocks under one minute. - There was a large variation in participants’ abilities during JTHF test. One participant took shorter time to complete the small object task while another took longer for the same task while both using the orthosis. - Pinch force test showed that two out of three individuals increased the strength of their three-jaw chuck grasp and the key grip was more natural. |
| **Kang *et al.* (2013)** | - Three-point pinch force - MVC of wrist extension | - Average pinch force for all participant was 0.64 ± 0.42 N. - The manual muscle test (MMT) was scored at least grade 3 for all subjects. - Wrist extension muscle voluntary contraction ranged from 1.92 Nm at 29.4° to 0.46 Nm at 26.4°. | - Pinch force was 7.26 ± 3.48 N which is 14.3 times greater than pinch force without the orthosis. - Greater MVC was recorded which resulted in a greater pinch force. |
| **King *et al.* (2009)** | - Preston pinch meter - Thirteen ADLs (remote control, card in and out, fork and putty, key in and out, key Turn, horizontal zip open and close, vertical zip open and close, and electric plug in and out) | - Wrist extension strength was greater than grade 3 and thumb flexion was less than grade 3. - Lateral key grip force without orthosis was 4.3 N (range 2.0–8.0 N). - Limited ADL tasks. | - Participants increased lateral key grip force and achieved an average force of 13.1 N (range 4.7– 22.3 N). - Greater number of ADL tasks achieved with orthosis. |
| **Rohm *et al.* (2013)** | - FES-generated GRT - Three ADLs (pick up a pretzel stick to eat, writing task, eating an ice cream cone) | - Shoulder: Active abduction, extension and flexion up to 30°. All grade 3/5. Full passive ROM. - Elbow: No active flexion (biceps grade 0/5, brachioradial muscle grade 0/5), no active extension (triceps grade 0/5). Full passive ROM. - Forearm: no active supination (grade0/5). No active pronation (grade 0/5). Full passive ROM. - Wrist, thumb and fingers: No active movements (grade 0/5). Almost full passive ROM in finger joints, full wrist and thumb ROM. | - Ability to successfully perform GRT tasks. Within trails of one minute, the participant succeeded transferring Double blocks (7 completions out of 7 attempts) and Pegs (10 completion out of 18 attempts) over the frame of a box. |
| **Varoto *et al.* (2008)** | - Rotation speed - Real-time angular variation - Real-time force measurement | - Shoulder: Active movements of the shoulder and scapula. - Wrist, thumb and fingers: Limited active grasping functions. | - Combining shoulder and scapula movements with the increased ability to manipulate objects using palmar grasp. |
| **Cappello *et al.* (2018)** | - TRI-HFT to identify ability to manipulate objects and weights that would be encountered during ADL. This test is divided into three parts:  1. Ten ADLs to manipulate objects using palmar and pinch grasps. 2. Strength test – qualitative test using weighted objects. 3. Strength test – quantitative test using a hand-held dynamometer. | - Limited hand function, specifically strength and range of motion. - TRI-HFT average performance of 53.88 ± 24.20% was recorded. - Average lift force of 1.76 ± 4.32 N was recorded. | - Average score of 87.30 ± 11.82% on TRI-HFT. - The device provided a very firm and reliable palmar grasp, however improvement in pinch force was limited. - Mean lift force improved across all participants and a mean force of 2.76 ± 5.18 N was achieved. |
| **Asai & Kuroiwa (1999)** | - One ADL (consumption of yogurt over a period with a tablespoon). | - Inability of participants to perform a predefined ADL (namely eating yoghurt) independently. | - Three out of four of the participants consumed yogurt more easily with both (portable spring balancer (PSB) and mobile arm support (MAS)) devices. - All participants, except for one, showed larger mean consumption with PSB than MAS. The mean scores for one participant was inconsistent as it was initially reported not different between the two orthosis, but after repeating the tests the consumption using MAS almost doubled compared to using PBS. - Gradual improvement in task performance was noted as subjects demonstrated a positive uniform trend in both phases of using the PSB and MAS |
